# Scleral structural alterations associated with chronic experimental intraocular pressure elevation in mice

**Published:** 2013-09-26

**Authors:** Elizabeth Cone-Kimball, Cathy Nguyen, Ericka N. Oglesby, Mary E. Pease, Matthew R. Steinhart, Harry A. Quigley

**Affiliations:** Glaucoma Center of Excellence, Wilmer Ophthalmological Institute, Johns Hopkins University, Baltimore, MD

## Abstract

**Purpose:**

To study changes in scleral structure induced by chronic experimental intraocular pressure elevation in mice.

**Methods:**

We studied the effect of chronic bead-induced glaucoma on scleral thickness, collagen lamellar structure, and collagen fibril diameter distribution in C57BL/6 (B6) and CD1 mice, and in collagen 8α2 mutant mice (Aca23) and their wild-type littermates (Aca23-WT) using electron and confocal microscopy.

**Results:**

In unfixed tissue, the control B6 peripapillary sclera was thicker than in CD1 mice (p<0.001). After 6 weeks of glaucoma, the unfixed CD1 and B6 sclera thinned by 9% and 12%, respectively (p<0.001). The fixed sclera, measured by electron microscopy, was significantly thicker in control Aca23 than in B6 or CD1 mice (p<0.05). The difference between fresh and fixed scleral thickness was nearly 68% in untreated control B6 and CD1 mice, but differed by only 10% or less in fresh/fixed glaucoma scleral comparisons. There were 39.3±9.6 lamellae (mean, standard deviation) in control sclera, categorized as 41% cross-section, 24% cellular, 20% oblique, and 15% longitudinal. After glaucoma, mean peripapillary thickness significantly increased in fixed specimens of all mouse strains by 10.3 ±4.8 µm (p=0.001) and the total number of lamellae increased by 18% (p=0.01). The number of cellular and cross-section lamellae increased in glaucoma eyes. After glaucoma, there were more small and fewer large collagen fibrils (p<0.0001). Second harmonic generation imaging showed that the normal circumferential pattern of collagen fibrils in the peripapillary sclera was altered in significantly damaged glaucomatous eyes.

**Conclusions:**

Dynamic responses of the sclera to experimental mouse glaucoma may be more important than baseline anatomic features in explaining susceptibility to damage. These include decreases in nonfibrillar elements, alterations in lamellar orientation, an increased number of smaller collagen fibrils and fewer larger fibrils, and relative increase in the number of scleral fibroblast layers.

## Introduction

The structure of the cornea and sclera of the eye are instrumental in the translation of force generated by intraocular pressure (IOP) to the optic nerve head region, a principal site of damage to retinal ganglion cells (RGCs) in glaucoma [1,2]. The sclera comprises over 3/4 of the surface area of the human eye and its biomechanical response to IOP is of interest in the study of the pathogenesis of glaucoma. The sclera is derived largely from the neural crest and is 75–90% collagen by dry weight [3], mostly collagen type 1 [4], with variable amounts of other collagen species, elastin fiber, and proteoglycans. The normal scleral thickness varies by region in rodent [5], monkey [6], and human eyes [7,8]. Fibril diameter for scleral collagen is larger [9] and varies more than in the cornea [10] or the lamina cribrosa of the optic nerve head [11], each of which have uniform collagen fibril diameters. In addition to fibrillar molecules, there is an extensive complement of proteoglycans in the sclera, including heparin sulfate, chondroitin sulfate, dermatan sulfate, keratan sulfate, hyaluronan [12], aggrecan, and several small leucine-rich proteoglycans [13]. These may have important functional significance for the mechanical responses of the sclera.

The scleral collagen fibrils are arranged in fibril groups called lamellae in which most fibrils course parallel to each other, but with the dominant orientation alternating in successive lamellae through the scleral width. While electron microscopy suggests little interweaving between corneal or scleral lamellae, recent improvements in microscopy have clarified that individual fibrils interlace through multiple levels of the corneal stroma [14], and perhaps do so in the sclera. It is likely that there is interweaving of fibers much like the components of a woven basket that pass behind and in front of each other. Such construction could affect the mechanical behavior of the sclera with changes in stress differently from a structure in which layers do not intermingle in the anteroposterior plane.

There is a variety of orientations for the lamellae, with alternating lamellae having mostly anteroposterior, circumferential, and oblique fibrils. The exceptions to this variable orientation are the zones near muscle insertions, areas of the outermost and innermost scleral surface [15], at the limbus [16] and in the peripapillary zone. In the peripapillary sclera, collagen and elastin fibrils are oriented circumferentially around the human optic nerve head [17-20], and similarly in mouse [21], rat [22], and monkey [23] eyes. This would reinforce the peripapillary sclera at the location adjacent to the optic nerve head, at which there is a dramatic increase in strain induced by changes in IOP. Collagen and elastin fibrils within the lamina cribrosa in monkey and human eyes run directly across the canal from side to side [17].

The human scleral microanatomy differs with age, and in diseases such as myopia [4,24,25] and glaucoma [8,20,26]. Animal models mimic important features of human diseases with regard to scleral structure and remodeling. There is well known scleral alteration with experimental defocus and form deprivation in the guinea pig [27], tree shrew [28], and mouse [29]. Investigations of experimental glaucoma both in monkeys [30] and mice [31] have suggested that both the baseline scleral structure and its dynamic response may be important determinants of glaucomatous damage. We found that a mutation in collagen 8α2, a component of the cornea and sclera [32], leads to protection against injury in experimental murine glaucoma [33]. The present investigation seeks to determine the detailed structure and ultrastructure of the mouse sclera, its response to chronic IOP elevation, and scleral differences among three strains of mice that may correlate with their susceptibility to glaucoma.

## Methods

### Animals

All animals were treated in accordance with the Association for Research in Vision and Ophthalmology Statement for the Use of Animals in Ophthalmic and Vision Research, using protocols approved and monitored by the Johns Hopkins University School of Medicine Animal Care and Use Committee. Three different strains of mice, 2–4 months of age at the start of experiments, were studied: CD1 (Charles River Laboratories, Wilmington, MA), C57BL/6 (B6, Jackson Laboratories, Bar Harbor, ME), and Aca23 mutant mice rederived in B6 from heterozygotic Aca23 mouse embryos graciously provided by Drs. Puk and Graw [34]. The Aca23 mice are homozygous for a G257D exchange (Gly to Asp) missense mutation in the collagen 8A2 gene and have significantly longer and wider eyes than wild-type (WT) littermates, as well as greater resistance to experimental glaucoma damage [33]. In this study, 254 mice ranging between 3 and 5 months of age were used for axial measurements, and measurement of sclera thickness in fresh unfixed tissue (126 CD1 and 128 B6). Twenty animals were used to study axon loss, axial measurements, scleral thickness, and lamellar orientation in fixed tissue (five CD1, five B6, five Aca23 homozygous [Aca23], and five Aca23 littermate controls [Aca23-WT]). An additional nine animals were used to study the collagen orientation around the peripapillary region using Second Harmonic Generation (five 4-month-old CD1 and four 13-month-old CD1 mice), for a total of 283 mice.

### Anesthesia

For anterior chamber injections to produce elevated IOP, mice were anesthetized by either an intraperitoneal injection of 50 mg/kg of ketamine (Vedco Inc, Inverin Ireland), 10 mg/kg of xylazine (LLOYD Labs, Shenandoah, IA), and 2 mg/kg of acepromazine (Boehringer Ingelheim, St. Joseph, MO; during surgical procedures) or by inhalation of isoflurane (for IOP measurements only). For the latter, we used the RC^2^- Rodent Circuit Controller (VetEquip, Inc., Pleasanton, CA). This instrument supplies oxygen from an attached tank at 50–55 pounds per square inch. Oxygen is mixed with isoflurane and sent to two outflows at 500 ml/minute, delivering 2.5% of isoflurane in oxygen to the animal. One outflow entered a box where mice were placed for initial sedation. After about 2 min, the sedated animal was positioned for IOP measurement and clinical examination with a nose cone delivering the isoflurane gas/oxygen mixture. The nose cone permitted access to the eyes. Animals that were anesthetized with isoflurane did not receive topical anesthesia. Animals under ketamine/xylazine/acepromazine anesthesia received topical anesthesia of 0.5% proparacaine hydrochloride eye drops (Akorn Inc., Buffalo Grove, IL). We decided to use gas anesthesia because IOP readings were closer to awake IOP (in B6 mice), and to help decrease the morbidity from intraperitoneal injections, specifically in B6 mice [35].

### Bead injection protocol

Animals received 4 µl of a mixture of 6 µm and 1 µm beads (2 µl of 6 µm beads and 2 µl of 1 µm beads) followed by 1 µl of viscoelastic solution (10 mg/ml sodium hyaluronate; Healon, Advanced Medical Optics Inc., Santa Ana, CA) delivered through a glass cannula 50 µm in diameter connected by polyethylene tubing to a Hamilton syringe (Hamilton Company, Reno, NV), our “4+1” protocol [35]. The glass cannula was kept in place for 2 min to prevent the egress of beads after withdrawal.

### Intraocular pressure measurement

All IOP measurements were made using the TonoLab tonometer (TioLat, Inc., Helsinki, Finland), recording the mean of six readings with optimal variability grade. On bead-injected animals we measured IOP between 9 and 11 a.m. at baseline (before injection) at 1 day, 3 days, 7 days, and 14 days post bead injection. Prior to IOP measurement, animals were anesthetized by one of two methods—intraperitoneal injection or inhalation of isoflurane, as described above. We calculated the mean IOP over 6 weeks and two measures of cumulative IOP exposure, specifically the total difference between treated and control eye (total integral IOP) and the positive difference between treated and control eye (positive integral IOP), both in units of mm Hg—days.

### Axial length measurement

#### Fixed tissue:

Twenty mice (5-CD1, 5-B6, 5-Aca23-WT, 5-Aca23) that were intended for transmission electron microscopy studies received an intraperitoneal injection of general anesthesia before sacrifice by exsanguination, followed by intracardiac perfusion with 4% paraformaldehyde in 0.1 M sodium phosphate buffer (Na_3_PO_4_, pH=7.2). Eyes were then enucleated and a needle connected fluid-filled reservoir was inserted to produce an IOP of 15 mmHg to standardize measurement of the axial length and width. The measurements were performed with a digital caliper (Instant Read Out Digital Caliper, Electron Microscopy Sciences, Hatfield, PA). The length was measured from the center of the cornea to a position just temporal to the optic nerve, and both nasal-temporal width and superior-inferior width were measured at the largest dimension at the equator, midway between cornea and optic nerve. Some eyes underwent measurements of axial length and width as above after sacrifice under general anesthesia, but were never fixed. Axial length increase (“percent longer”) was calculated by taking the difference between the control axial length and the experimental axial length, then dividing by the control axial length.

#### Fresh tissue:

We measured axial length and width in “fresh tissue” in 126 CD1 and 128 B6 mice. To do this, we enucleated the eyes and placed them into sodium phosphate buffer (Na_3_PO_4_ pH=7.2). We measured the length and width of the eye using a digital caliper (following the same protocol as above) but without puncturing the eye with a needle connected to a fluid-filled reservoir.

### Retinal ganglion cell axon counts

To assess RGC damage in the 20 animals that were perfusion fixed (5CD1, 5B6s, 5Aca23-WT, and 5Aca23), we estimated axon loss in optic nerve cross-sections using a quantitative, random sampling technique [36]. The optic nerves were removed from the globes, placed in 1% osmium, dehydrated in ascending alcohol concentration, and then placed in 1% uranyl acetate in 100% ethanol for 1 h. Tissues were embedded in an epoxy resin mixture at 60 °C for 48 h. One micron thick cross-sections of the optic nerve were cut, stained with toluidine blue, and digital images of the nerves were taken at low power to measure each optic nerve area. Then, high power images were taken (100X, oil immersion objective) using a Cool Snap camera and Metamorph Image Analysis software (Molecular Devices, Sunnyvale, CA).

For each nerve, five 40×40 μm fields were acquired, corresponding to a 9% sample of total nerve area. Masked observers edited nonaxonal elements from each image, generating an axon density from the software. The average axon density/mm^2^ was multiplied by the individual nerve area to estimate the axon number. Experimental eyes were compared to the mean axon number in pooled, fellow eye nerves of the appropriate mouse strain, age, and tissue fixation to yield percent axon loss.

### Microscopic sclera measurements

#### Fresh tissue

**Figure 1:**
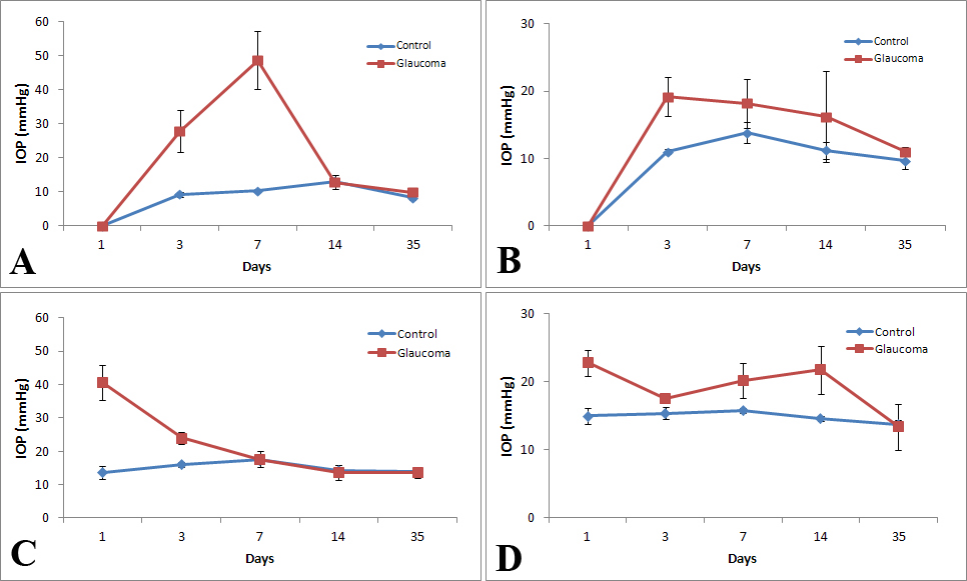
Mean intraocular pressure in control and glaucoma groups by strain. The mean intraocular pressure (IOP; mmHg) readings of the control eye and glaucoma treated eyes at five different time points post bead injection are shown in the panels as follows: **A**: B6, n=5; **B**: CD1, n=5; **C**: Aca23-WT, n=5; and **D**: Aca23, n=5.

We measured scleral thickness in 126 CD1 and 128 B6 mice, which we referred to above as “fresh tissue” (see Axial length measurement). The superior quadrant of fresh, unfixed sclera was cut from the limbus to peripapillary area and placed in buffer. Three strips measuring 0.33 mm wide and 2.5 mm long were cut from the peripapillary area (Figure 1A) to the limbus with a sharp blade. Each strip was measured at 6 locations from peripapillary (R1) to limbus (R6), every 0.5 mm (Figure 1A). Using an eyepiece micrometer, the mean of three measurements of scleral thickness was then determined in each of the six regions of the three strips from one eye and an overall mean was calculated. Parallel measurements done on fresh, unfixed scleral segments by confocal microscopy showed that the thickness obtained was consistent between the two methods [5] (data not shown).

#### Fixed tissue

In perfusion “fixed tissue” (4% paraformaldehyde, see Axial length measurement above), we studied scleral thickness, lamellar orientation, and fibril diameter distribution in peripapillary, nasal, and temporal sclera of 20 animals in both the glaucoma eye and the fellow control eye, with five mice from each strain, specifically CD1, B6, Aca23-WT, and Aca23. For transmission electron microscopy (TEM), three zones were studied in detail: the superior peripapillary region, and two regions approximately 0.5 mm anterior to the nerve head border (at Region 2/3, Figure 1A), one nasal and one temporal (see Figure 1B). After tissue was oriented with the superior due north, a trephine was used to remove the optic disc (Figure 1B2). Using a razor blade, the tissue was cut flush at the superior border of the optic nerve head, where sectioning was begun to maintain orientation. Strips from the peripapillary area to the limbus were made using razor blades (Figure 1B4) in both the temporal and nasal regions. Each strip was divided into two pieces, one containing Region 2/3 and the other containing Region 4 (Figure 1B6). Diagonal cuts were made at the corner of each tissue to help in orientation when embedding the tissue in epoxy resin (Figure 1B6). Fixed tissue was epoxy processed following the same protocol used to embed optic nerves (see Retinal ganglion cell axon counts).

Ultrastructural scleral measurements: Ultrathin sections (48–68 nm) were taken from representative areas and placed on copper mesh grids (Electron Microscopy Sciences), stained by a 0.01% uranyl acetate and 0.57% tannic acid solution followed by the addition of 0.3% Reynolds lead citrate solution before TEM examination (Hitachi H7600; Hitachi High-Technologies Corp., Tokyo, Japan). Digital images of the sclera were taken at three locations in each regional sample. At each location, an initial, low-magnification image (3,000–4,000X) was taken to measure overall scleral thickness, followed by a sequence of four to seven images (10,000–12,000X) to assess the scleral lamellae according to their number, thickness, and classification into one of the four category types. While examining the first cross-section of the sclera (in the peripapillary zone), we defined the directions of fibers in lamellae as oriented in one of three directions. Using the Metamorph Software System, we were able to measure the shape of the collagen fibrils while using a shape ratio. This tool measures the shape of individual fibrils, compares them to a circle, and gives a ratio to help determine the shape of the object. When quantifying objects (in this case the collagen fibrils), those closer to 1 are considered more circular and ratios closer to 0 are less circular. In our study, we only used collagen fibrils with a shape ratio of 0.9 or greater. The orientation of these fibrils will be referred to as cross-section (corresponding to anterior-posterior direction). Second, a lamella was designated longitudinal if most of its fibers ran from one side to the other of the viewing plane as a continuous structure (parallel to the plane of the section and corresponding to circumferential direction around the eye). Those that did not fall into either of these categories were called oblique.

**Figure 2:**
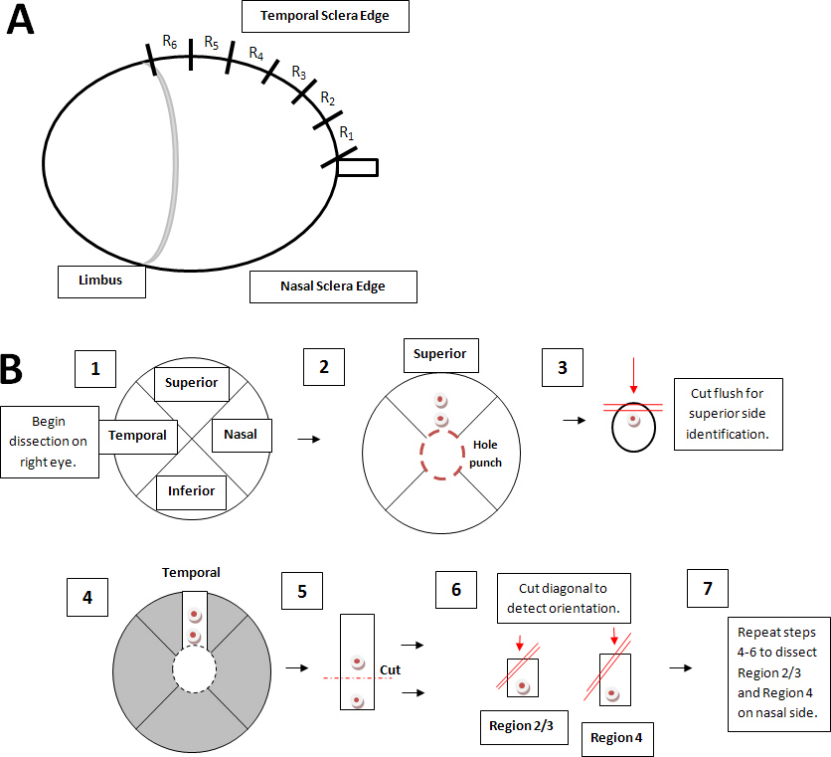
Scleral tissue division. The method of identifying areas of the sclera for measurement of scleral thickness, lamellar orientation, and fibril diameter distribution is shown. **A**: For sclera thickness measurements in fresh tissue slices, the sclera was divided into six regions, labeled R1 (peripapillary) to R6 (limbus). **B**: The sclera was divided into the following pieces; (1) Four zones; superior, nasal, inferior, and temporal. (2) The optic nerve head and peripapillary sclera were removed with a 1.5 mm in diameter hole punch. (3) Razor cuts were made at the superior to assist with identification during plastic embedding and sectioning. (4–7) From each quadrant, two specimens (representing Regions 2+3 and Region 4 in A above) were selected for epoxy embedding, as was the peripapillary specimen.

The final and fourth category of lamellae was noncollagen material, and this comprised cellular components (Figure 2). We measured the thickness of each lamella and calculated the proportion represented by each type in the full scleral thickness of each region.

The collagen fibril diameter was measured in the peripapillary region from 10 to 12 zones (40,000X) in which the fibrils were oriented in cross-section. We used the Metamorph Image Analysis software (Molecular Devices, Downington, PA) to measure the diameter of each fibril in an automated fashion. In these TEM 40,000X magnification images, collagen fibrils in cross-section have an outer membrane-like rim and a lighter inner zone. While the rim is thin, it has a finite thickness, so the software measures the diameter from the inner border of this zone from one side to the other. After the automated diameter sections were complete, we calculated the overall density and the average fibril density at each diameter from zero to 200 nm divided into bins of 2 nm width.

The variation in peripapillary scleral collagen fibril diameter across the scleral thickness was also measured in the four groups of mice—CD1, B6, Aca23-WT, and Aca23. Three images were taken within each of the following three scleral regions: inner (close to the choroid), mid-sclera, and outer sclera. As mentioned above, we used the thin dark rim around the fibril as a way to measure the diameter of each collagen. Using a line tool, a masked observer individually measured 100 fibrils (from one side of a fiber to the other) at each of the three locations (inner, mid-, and outer sclera) in all four mouse strain groups. The Metamorph Imaging software then converted the measurements from pixels to nanometers. To check for repeatability, the masked observer tested 300 fibrils in 20 different images and found that the average mean differed by 2.6%, a figure suitable for reproducibility.

### Second harmonic generation imaging

Second harmonic generation (SHG) is a nonlinear imaging method that identifies collagen molecules [37] by the stimulation of an excitation wavelength and detection of the emission of photons at half the excitation wavelength. This technology permits evaluation of the fresh, unfixed whole sclera and optic nerve head without embedding or sectioning. For example, excitation of collagen at 780 nm will generate emission at 390 nm without the need for added dyes or antibodies. SHG photons typically propagate in the direction of the excitation beam (transmitted signal). Advantages of this image modality include less scatter and better penetration than conventional single photon imaging. The wave characteristics of SHG also eliminate the need for a pinhole, resulting in better signal collection at lower power settings. The resulting higher energy signal is collected from deeper structures, allowing high resolution, three-dimensional imaging [38].

A Zeiss LSM 710 nonlinear optics, multiphoton microscope (Zeiss, Oberkochen, Germany) with a Chameleon Ultra II, mode locked, titanium:sapphire tunable laser (680–1080 nm; Coherent Inc., Santa Clara, CA) and non-descanned detector was used. The multiphoton laser delivers 80 MHz, 140 femtosecond pulses in the near infrared. Forward-scattered SHG was collected using a high numerical aperture condenser and the non-descanned detector with LP490/SP480. Unfixed scleral flat mounts from eyes of both control and glaucoma groups were prepared at 1 mm x 1 mm in size. These had the optic nerve head in their center and the optic nerve removed at the posterior border of the sclera. The sclera was initially scanned at 20× (Plan-Apochromat 20X/0.8), using 2,048 resolution, 0.6 zoom, averaging two images with 40–70 slices, 1 µm apart, in 2×2 tiles as z-stacks. For analysis at higher power, 2×2 z-stacks were acquired using 40× (Plan-Apochromat 40X/1.4 Oil DIC M27) at 1,772 resolution, and 1.0 zoom. Each slice was an average of two images per section, 0.5 µm apart, for a total of 80–140 slices.

Eighteen posterior scleral specimens were examined by SHG from nine mice, each of which underwent the chronic glaucoma model for 6 weeks in one eye; they consisted of five 5-month-old CD1 mice and four 13-month-old CD1 mice. Both the glaucoma eye and fellow control eye were examined by SHG imaging to evaluate the collagen structure in the posterior 2 mm of the sclera, including the optic nerve exit area. After initial observations showed a likely difference between control and glaucoma, a masked observer examined each and graded them as either glaucoma or control based on changes in the peripapillary scleral collagen that are detailed below.

For each of the 18 eyes, epoxy embedding and sectioning were completed as stated above in the Retinal ganglion cell axon counts section. These optic nerves were not counted using Metamorph imaging software, since their fixation by immersion does not provide adequate preservation for automated assessment. Instead, they were graded by a masked observer for the amount of axon loss on a 4-level semiquantitative scale by a masked observer for the amount of axon loss as follows: 1=normal, 2=10–30%, 3=30–65%, and 4=65–100%.

### Statistical analysis

For outcome variables measured on each eye, such as axial length, axial width, scleral thickness and lamella count, means and standard deviations were calculated by treatment and mouse strain. Here, student *t*-tests and paired *t* tests were used to make unadjusted comparisons. The effect of the chronic glaucoma treatment on outcome in four mouse strains was explored using a general linear mixed model, taking into account the correlation in outcome between a mouse’s control eye and glaucoma eye. Two models were fit for each outcome. The first looked at the effect of treatment adjusted for strain, while the second included a term for the interaction between treatment and strain to identify strains for which there was a difference in outcome in control eyes and strains for which there was a difference in the effect of glaucoma treatment. Bonferroni adjusted p values were used for multiple comparisons. Unadjusted p values were used to compare strains Aca23-WT and Aca23. To measure the effect of glaucoma on percent of total thickness in a zone represented by a particular lamellar type by strain, the percentages were transformed using the logit function (natural logarithm of p/(1-p)) and were analyzed using the same method as above. For outcome variables measured on a mouse, such as average IOP difference, positive and total IOP integral and axon loss, the distribution of the variable was examined to see whether the assumption of normality was reasonable. Medians as well as means and standard deviations were calculated. Since average IOP difference appeared to be normally distributed, a *t* test was used to evaluate the significance of the overall difference and analysis of variance was used to look at the effect of strain. Positive and total IOP integral and axon loss did not appear to be normally distributed. A sign test was used to evaluate overall significance and the Kruskal-Wallis test was used to look at the effect of strain. Fisher’s exact test was used to compare the classification of eyes as control or glaucoma from laboratory findings to their true status. For figures, general linear models were used to obtain regression lines and R-square statistics. All analyses were performed using SAS 9.2 (SAS Institute, Cary, NC).

## Results

### Elevation in intraocular pressure with glaucoma

The mean difference in IOP between glaucoma and fellow eyes was 6.2+4.4 mmHg (p<0.0001, *t* test; Figure 1). Overall, the positive integral and total integral IOP difference were higher in glaucoma than in fellow eyes (p=0.01, 0.0004, respectively). The different mouse strains did not differ in the degree of IOP increase with glaucoma, judged by mean IOP difference or positive and total integral IOP differences (p=0.13–0.18, general linear regression model–Tukey adjustment for multiple comparisons).

### Axial length and width and retinal ganglion cell axon loss

The axial length and width of the four mouse strains differed in control, untreated eyes, with Aca23 having the greatest length at baseline, 3.74±0.15 mm, and B6 the smallest length, 3.34±0.05 mm (p=0.0005, *t* test; Table 1), duplicating our prior data on these mouse strains [33]. The axial length and width significantly increased after glaucoma overall, with a mean length in glaucoma eyes of 3.81±0.43 mm compared to 3.50±0.21 mm in controls, and width in glaucoma of 3.49±0.31 mm compared to 3.32±0.21 mm in controls. This comprised enlargements of 9% and 5%, respectively, in length and width (p=0.001, 0.03, respectively; *t* test, n=40). The Aca23-WT length and width increase after glaucoma was significantly greater than that in B6 (p=0.0001, *t* test), and more than in Aca23 or CD1 (p=0.001, *t* tests). The CD1 and Aca23 length and width increase with glaucoma was significantly greater than that of B6 (p=0.01, *t* test).

**Table 1:** Ocular length and width

**Strain**	**Length (mm)**		**Width (mm)**		**% longer**
	**Control**	**Glaucoma**	**Control**	**Glaucoma**	
**All**	**3.50±0.21**	3.81±0.43*	**3.32±0.21**	**3.49±0.31^+^**	8.9%
**Aca23**	3.74±0.15	4.04±0.43	3.50±0.19	3.70±0.10	8.0%
**Aca23-WT**	3.46±0.26	4.08±0.28	3.32±0.26	3.70±0.14	17.9%
**B6**	3.34±0.05	3.37±0.38	3.19±0.05	3.09±0.25	0.9%
**CD1**	3.46±0.09	3.75±0.29	3.28±0.19	3.46±0.23	8.4%

n=5 eyes in each category (fixed tissue); mean ± standard deviation

Aca23-WT and CD1 animals exhibited the greatest amount of axon loss (median 31% and 26%, respectively). B6 nerves had a median of 11% loss, while Aca23 mutant animals showed no significant axon loss (median=3% greater than control axon count). The axon loss was significantly affected by strain of mouse (Kruskal-Wallis, p=0.05). Aca23-WT axon loss was significantly greater than Aca23 (p≤0.02, *t* test). B6 mice exhibited less axon loss than CD1 ones, but the difference was not significant with the sample size in this study (p=0.39, *t* test). In previous published research using larger sample sizes, B6 mice were significantly less susceptible to axon loss than CD1 mice [35].

### Scleral thickness in fresh tissues

We evaluated scleral thickness according to two conditions, specifically in fresh, unfixed scleral slices [5] and in TEM thin-sectioned images after fixation and epoxy embedding. In control eyes, the unfixed sclera was thicker at the peripapillary zone than in the adjacent regions (R2, 3; p<0.0001, *t* test, Table 2). With glaucoma, the peripapillary sclera became significantly thinner (9–12%) in both CD1 and B6 strains (Table 2). In CD1 mice, zones R2 and R3 also thinned significantly after glaucoma, but in B6 mice, regions R2 and R3 were actually thicker than in controls, although the increase was not statistically significant (R3, p=0.06).

**Table 2:** Scleral thickness, unfixed tissues

**Strain**	**Treatment**	**N**	**Peripapillary**	**Region 2**	**Region 3**
B6	Control	128	61.1±8.0	47.7±6.8	41.0±6.3
B6	Glaucoma	42	55.6±9.8*	48.4±7.6	43.4±9.1
CD1	Control	126	55.1±7.7	46.0±7.1	40.4±6.0
CD1	Glaucoma	43	48.7±9.0*	41.1±6.9*	36.8±6.5*

Data are in µm; mean ± SD *glaucoma mean significantly lower than control, p≤0.001, *t* test.

### Scleral thickness and lamellar structure, fixed tissue

#### Peripapillary sclera

**Figure 3:**
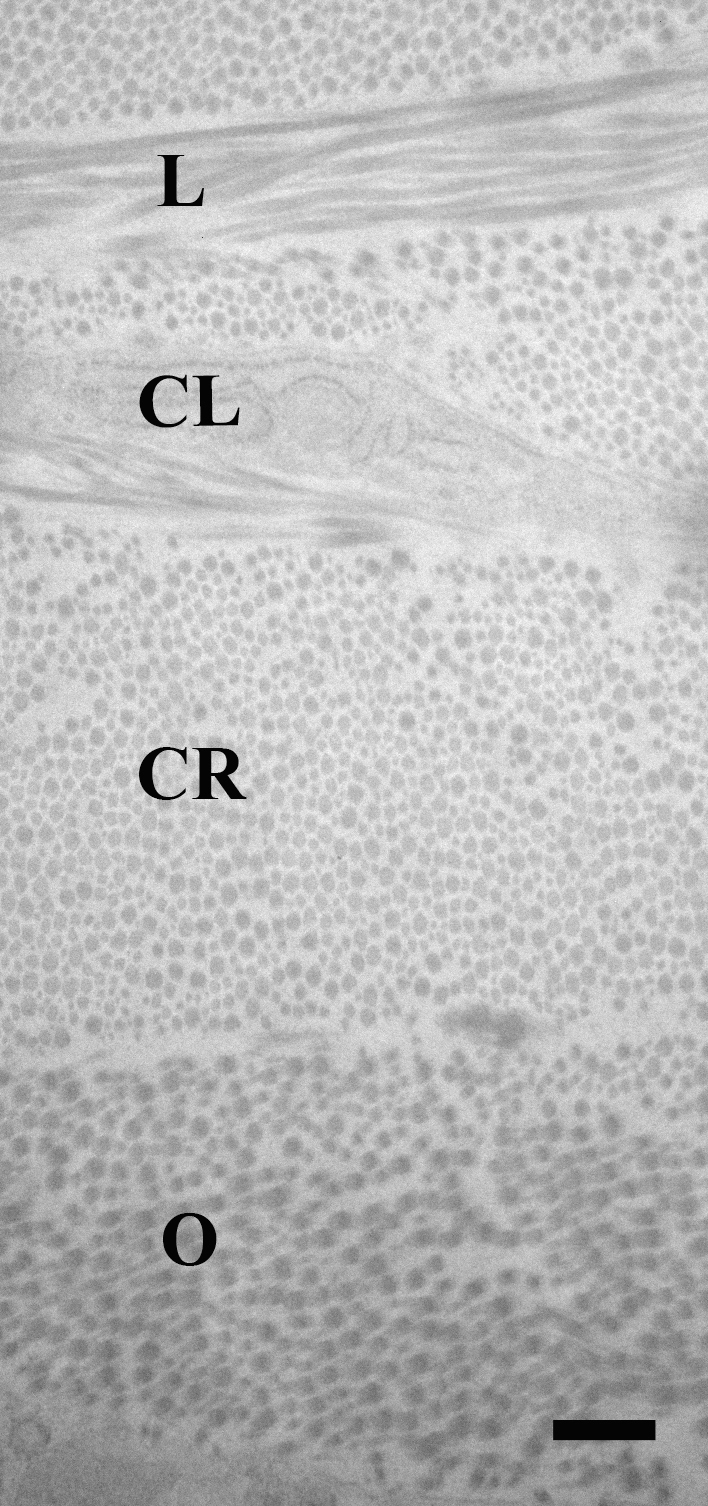
Collagen lamella orientation. Using transmission electron microscopy (TEM), the lamellae of the sclera were quantified and their thickness measured by dividing them into the following four types based on their orientation of collagen fibrils: cross-section (fibrils anterior-posterior=CR), longitudinal (parallel to equator, nasal-temporal=L), oblique (O), and lamellae consisting of sclera fibroblasts (cellular=CL; scale bar=500 nm).

In the peripapillary region (Figure 2B3), mean total scleral thickness across all four types of control mice was 42.5+10.5 µm; however, the peripapillary sclera was significantly thicker in Aca23 mutants than in B6 or CD1 (p<0.05, general linear mixed model, Bonferroni correction, Table 3, and Appendix 1). Aca23 mice did not differ significantly from Aca23-WT littermates (p>0.05). These scleral thicknesses, measured in fixed tissue with TEM observations, were thinner than in fresh, unfixed tissue measurements of comparable strains (above) by 40% in CD1 and by 35% in B6. There were, on average, 39.3±9.6 lamellae of all types from the choroid to external sclera in untreated mice, overall. The distribution of the four types of lamellae as a percentage of the whole sclera was 41% cross-section, 24% cellular, 20% oblique, and 15% longitudinal (see Figure 3). Both control Aca23 mutant and Aca23-WT mice had more lamellae of all types in this region than did control B6 and CD1, but these differences were not significant (p>0.05). The most striking differences among control mice were observed in the types of lamellar structure. The Aca23 mutants and Aca23-WT had significantly more cellular lamellae (12.7 and 14.4, respectively) compared to B6 or CD1 (6.6 and 6.6, respectively) mice (p<0.05, *t* test). The Aca23 mutants and Aca23-WT also had greater total thickness of cellular lamellae, 12.4±4.1 and 18.9±8.0 µm, respectively, than B6 or CD1 5.4±0.8 and 5.1±2.4 µm, respectively (p<0.05). Aca23 mutants had significantly greater total thickness of cellular lamellae than Aca23-WT littermates (p<0.05, *t* test). The only other peripapillary differences in lamellar detail in untreated eyes were significant differences between Aca23 and Aca23-WT, with the former having significantly greater total thickness of longitudinal lamellae, 7.6±2.5 and 4.5±2.1 µm, respectively, and fewer oblique lamellae, 7.4±2.6 and 9.8±1.1, respectively (p=0.05, 0.03; Appendix 1 and Appendix 2, Figure 3).

**Table 3:** Peripapillary Scleral Thickness, Fixed Tissue (Region 1)

**Strain**	**Total thickness (µm)**		**Glaucoma/** **Control Ratio**	**Number of Lamellae**		**Glaucoma/** **Control Ratio**
	Control	Glaucoma		Control	Glaucoma	
Aca23	54.3±8.0	63.4±13.6	1.17	45.5±7.8	57.1±1.2	1.25
Aca23-WT	46.5±6.0	50.7±8.0	1.09	46.1±10.4	47.8±5.3	1.04
B6	36.4±6.3	52.5±8.3	1.44	32.9±3.1	40.9±7.4	1.24
CD1	32.7±4.4	44.3±10.1	1.35	32.7±6.6	41.4±9.1	1.27

n=3 to 5 eyes per category

After glaucoma, the total mean scleral thickness in fixed, peripapillary sclera significantly increased in all mouse strains by 10.3±4.8 (15.8) µm (mean [confidence interval], p=0.001). Note that this differed from the measurements of fresh sclera above, in which scleral measurements were thinner after glaucoma. Furthermore, the difference between fresh and fixed scleral thickness was nearly 68% in untreated control B6 and CD1, but differed only by 10% or less in fresh/fixed glaucoma sclera comparisons (Table 4).

**Table 4:** Ratio between fresh and fixed sclera thickness.

**Strain**	**Fresh (µm)**	**Fixed (µm)**	**Ratio (Fresh/Fixed)**
B6 control	61.1	36.4	1.68
B6 glaucoma	55.6	52.5	1.06
CD1 control	55.1	32.7	1.69
CD1 glaucoma	48.7	44.3	1.1

There were 7.2±2.3 more total lamellae after glaucoma, or an 18% increase (p=0.01, linear mixed model). After glaucoma exposure, models assessing change in scleral thickness or number of lamellae showed that all three types of collagenous lamellae increased considerably in thickness (all p≤0.02), but the percent of thickness they occupied did not change. The thickness of cellular lamellae increased and the fraction it occupied increased by 19%, but these changes were of borderline significance (p=0.06). The number of both cellular and cross-sectioned collagen lamellae also increased significantly (p=0.01), but longitudinal and obliquely oriented lamellae did not change significantly in glaucomatous sclera.

When models were constructed to compare only the effect of glaucoma on Aca23 mutants and their WT controls, Aca23 had significantly higher numbers of cellular and cross-section lamellae (20.9 and 21.5) than Aca23-WT (11.1 and 14.8) in peripapillary sclera (both p=0.01), as well as fewer longitudinal lamellae than Aca23-WT (6.2 versus 8.9, p=0.04).

#### Nasal and temporal sclera

At baseline, the fixed tissue TEM measurements found that the mean nasal scleral thickness was 31.3±6.8 µm and mean temporal thickness was 32.3±5.3 µm, each about 25% thinner than the thickness at the peripapillary zone (Appendix 1). The proportion the scleral thickness, normally occupied by each of the four types of lamellae, at the nasal and temporal quadrants was similar to that in the peripapillary zone. The total number and thickness of the four types of lamellae in the nasal and temporal quadrants of the sclera also did not differ significantly when comparing the control mice of the four mouse strains. After exposure to glaucoma, the total scleral thickness in all mice overall increased at the nasal quadrant by 5.0±0.12 µm and temporally by 5.2±1.2 µm (p=0.05). In models comparing either all four mouse strains or comparing Aca23 to Aca23-WT, there were no significant differences in number, thickness, or percent occupied by individual lamellar types.

#### Qualitative observations of sclera

Detailed evaluation of TEM images did not indicate substantial change in the organization of lamellae. Nor did it change in the intercellular spaces of cellular layers that might represent evidence of new synthesis of fibrillar elements.

### Fibril diameter

#### By location

**Figure 4:**
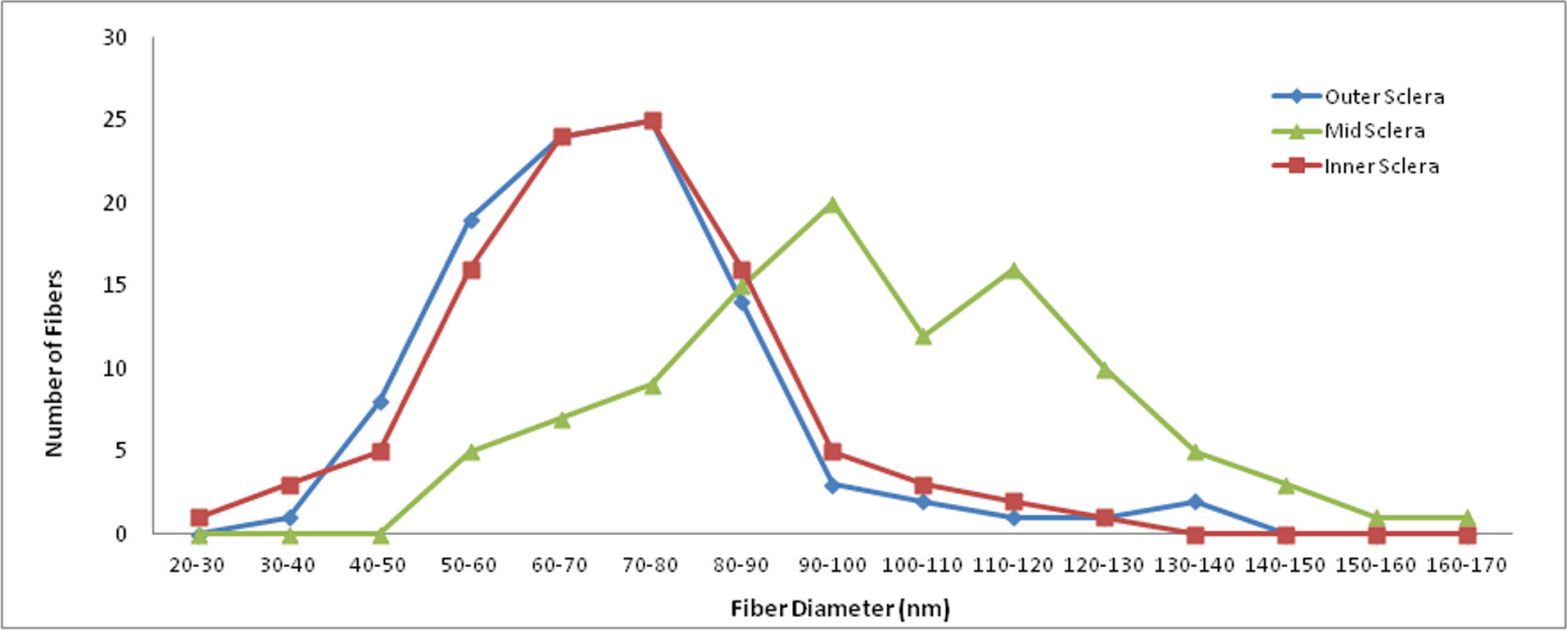
Collagen fibril diameter distribution by sclera location in CD1 control. The distribution for 100 collagen fibrils from a control CD1 mouse taken from the inner, mid-, and outer sclera shows that the mid-sclera has a broader range of fibril diameter and higher mean fibril diameter. The inner and outer sclera were different when compared to the larger fibrils found in the middle portion of the sclera (≤0.0001 and ≤0.0001 respectively, *t* test).The inner and outer scleral collagen diameter measurements, however, were not statistically different from one another.

In CD1 control peripapillary sclera, the mean collagen fibril diameter distribution of the middle scleral layers differed from that of the corresponding outer and inner sclera. The middle zone’s fibril diameter was 101.2±22.1 nm, while the outer and inner sclera had mean fibril thicknesses of 70.5±17.9 nm and 70.5±17.9 nm, respectively. The fibrils in the middle sclera were 42% thicker than the inner and outer sclera fibrils (both p≤0.0001, *t* test, n=100 fibrils per sclera-region). The middle sclera had 8–16 times more 110–120 nm fibrils than the inner or outer sclera (Figure 4). The inner and outer sclera diameter distribution did not differ.

#### By mouse strain

**Figure 5:**
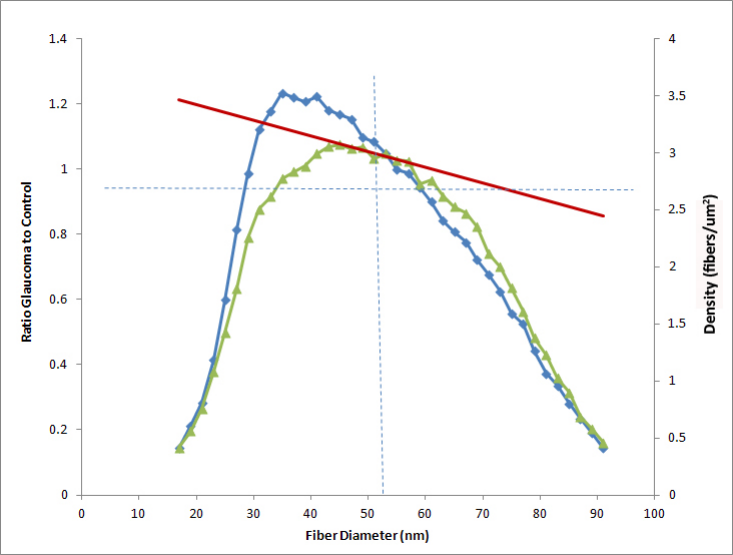
Collagen fibril diameter distribution: glaucoma compared to control. A histogram of collagen fibril diameter density (fibers/um^2^) is plotted for the control mouse sclera (green line, n=20 mice; 5 CD1, 5 B6, 5 Aca23-WT, and 5 Aca23) and glaucomatous sclera (blue line, n=19 mice; 5 CD1, 5 B6, 5 Aca23-WT and 4 Aca23; indicating density on the right y-axis). The ratio of the glaucoma density to control density at each diameter is represented as a linear regression (red line: r^2^=0.62, indicates values of ratio glaucoma to control, left y-axis). The glaucoma group has a higher proportion of smaller collagen fibrils and a lower proportion of larger fibrils with the crossover from more, smaller to fewer, larger fibrils occurring near the mean fibril diameter for controls (vertical dotted line).

Mean collagen fibril diameter in the peripapillary sclera was not significantly different in the various mouse strains (p≥0.12, *t* test). Control CD1 mice had the largest mean collagen fibril diameter of 55.0±8.8 nm, B6 mice had 54.9±7.0 nm, Aca23-WT had 49.5±4.4 nm, and the smallest diameter was in Aca23, 48.9±4.0 nm. After exposure to elevated IOP for 6 weeks, mean fibril diameter and density for all strains decreased slightly, but not significantly (p=0.25, p=0.29, regression model, Table 5). The collagen fibril diameter data were calculated as fibril per area (density, fibers/um^2^) and divided into bins that indicated density by size every 2 nm. We then calculated the ratio of glaucoma to control density in each bin and plotted that as a line in Figure 5). In our data analysis, we excluded bins containing fibers smaller than 19 nm and bins with fibers larger than 101 nm. The presence of these fibers varied within samples, but they represented less than 1.5% of all fibers found in the sclera. The regression line between the glaucoma/control ratio data only represented bins with large number of fibers, allowing the data to be weighted equally.

**Table 5:** Fiber Diameter and Density (fibers/1um^2^)

**Strain**	**Fiber Density**		**Fiber Diameter**	
	**Control (nm)**	**Glaucoma (nm)**	**Control (nm)**	**Glaucoma (nm)**
	Mean ± SD	Mean ± SD	Mean ± SD	Mean ± SD
Aca23-WT	90.0±14.8	92.2±13.7	55.0±8.8	52.0±4.7
Aca23	88.4±7.1	94.3±11.8	54.9±7.0	53.3±3.2
B6	74.2±15.0	75.6±8.0	49.5±4.4	50.6±2.7
CD1	70.8±14.1	79.8±10.7	48.9±4.0	42.7±8.6

**Figure 6:**
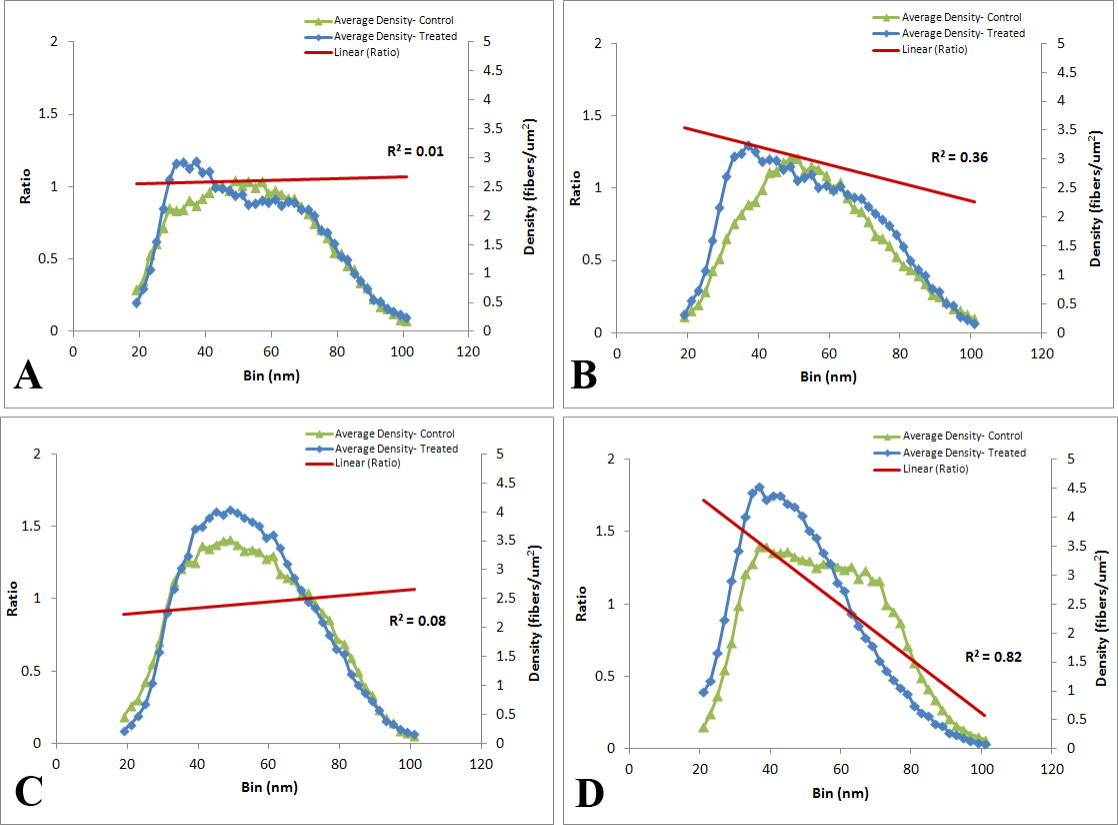
Regression lines and values for all four mouse strains. Histogram of collagen fibril diameter density (fibers/um^2^) is plotted for the control mouse sclera (green line) and the glaucoma sclera (blue line) for each mouse strain. The ratio of the glaucoma density to control density at each diameter grouping is represented as a linear regression (red line) for each of the four mouse strains as follows: **A**: B6 (n=5, R^2^=0.01); **B**: CD1 (n=5, R^2^=0.08); **C**: Aca23-WT (n=5, R^2^=0.36), and **D**: Aca23 (n=5, R^2^=0.82). The Aca23 and CD1 glaucoma groups had a higher proportion of smaller collagen fibrils and a lower proportion of larger fibrils compared to their controls (both p<0.0001).

It can be seen that glaucomatous mice exhibited a significant increase (ratio greater than one) in the groups of fibrils in bins smaller than the normal mean diameter and a significant decrease in the ratio in bins larger than the mean (Figure 5; linear regression, r^2^=0.62, p<0.001). When this glaucoma/control ratio by fibril diameter bin was evaluated in each mouse strain, the steepest slope (greater increase in small and decrease in large fibrils) was seen in Aca23 mutants (r^2^=0.82, p<0.0001, Figure 6D), followed by CD1 (r^2^=0.36, p=0.0001, Figure 6B), with no significant slopes for B6 (r^2^=0.01, p=0.8, Figure 6A) or Aca23-WT (r^2^=0.08, p=0.12, Figure 6C).

### Second harmonic generation collagen structure

In untreated fellow eyes, the collagen fibers in the zone immediately surrounding the optic nerve canal were oriented in a circumferential fashion. This extended from the canal opening outward for about 70–80 µm in width in the superior quadrant, 90–100 µm in the nasal and temporal quadrant, and about 60–70 µm in the inferior quadrant. In depth, the zone of circumferentially oriented fibrils were found most prominently near the interior sclera, nearest to Bruch’s membrane, and became more randomly oriented in the exterior sclera. From the circumferential zone outward, the fibril lamellae were arranged in a basket weave arrangement, often alternating at angles of 90 degrees or less and interweaving at very shallow angles on the Z axis (inner toward outer sclera).

**Figure 7:**
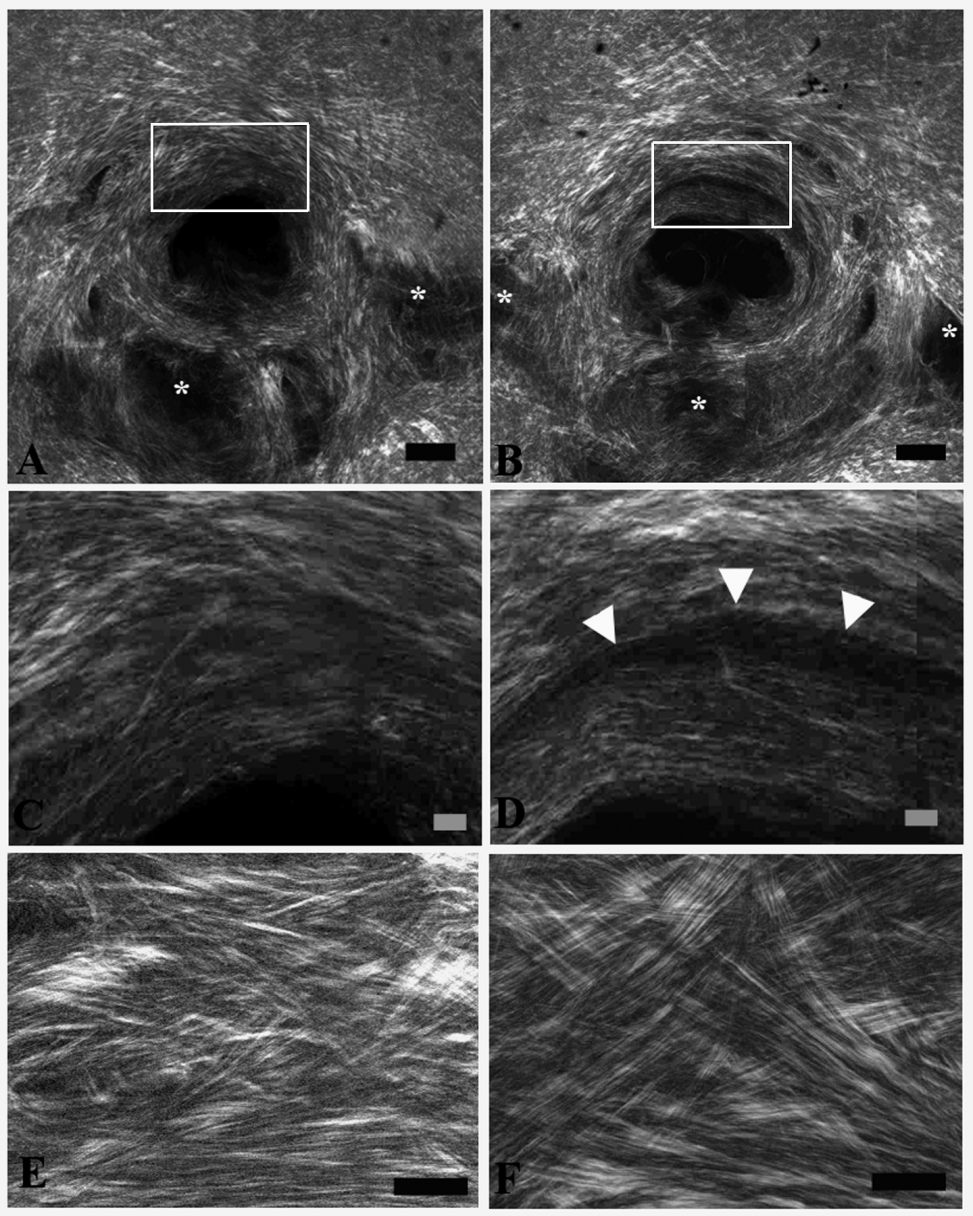
Second harmonic generation images. Second harmonic generation (SHG) imaging shows collagen orientation in the sclera of an older CD1 mouse; the 1 mm^2^ sclera piece includes the optic nerve canal (seen as the dark central area). The superior is located at the top of the images, while blood vessels (*) surround the optic nerve head on the nasal, inferior and temporal portion of the sclera. **A**: The peripapillary (posterior) sclera in control specimen had collagen fibrils oriented in a circumferential fashion around the optic nerve. **B**: In eyes exposed to elevated intraocular pressure (IOP), there was a retraction of this circumferential zone away from the optic canal, especially in the superior area. The white boxes in first two images (**A**, **B**) highlight the superior region. **C**: A 20× image of the posterior sclera shows no retraction in control tissue at the circumferential zone of the superior region. **D**: The retraction of collagen fibrils can be seen at the circumferential zone of the superior quadrant near the optic canal in 6 week glaucoma-treated eyes (20X). Moreover, 40× images of the mid-sclera show collagen assembled in a basket weave formation, where **E**: untreated tissue and **F**: glaucoma-treated tissue, which shows a slight change in fibril assembly. Scale bars are equal to 100µm for images **A** and **B**, and 10 µm for images **C**, **D**, **E**, and **F**.

In eyes exposed to elevated IOP, the circumferential zone was still present close to the choroid, but immediately behind this, the collagenous ring was found at greater distance from the optic nerve canal, especially in the superior region (Figure 7). The masked observer used this finding to correctly identify all eight specimens from older CD1 mice as either glaucomatous or untreated control tissue. Among the five younger CD1 mice, three glaucoma and three controls were correctly identified by this finding (overall, 14/18 correctly identified, p=0.057, Fisher’s exact test). Retraction of the border of the scleral canal as seen in SHG was found in all 5 mice with 30% or more glaucoma damage.

### Elevation in intraocular pressure with glaucoma and retinal ganglion cell axon loss

The mean difference in IOP between glaucoma and fellow eyes was 6.2±4.4 mmHg (p<0.0001, *t* test; Table 6). Overall, the positive integral and total integral IOP differences were higher in glaucoma than in fellow eyes (p=0.01, 0.0004, respectively, sign test). The different mouse strains did not differ in the degree of IOP increase with glaucoma, judged by mean IOP difference (p=0.18, analysis of variance) or positive and total integral IOP differences (p=0.13, 0.14, respectively, Kruskal-Wallis).

**Table 6:** IOP and axon loss

**Strain**	**Average IOP Difference, Glaucoma—Control (mmHg)**		**Axon Loss Compared to Pooled Control for Strain (%)**	
	**Mean ± SD**	**Median**	**Mean ± SD**	**Median**
Aca23	4.24±4.24	2.2	−4.3±12.3	−3.1
Aca23-WT	6.88±2.36	7.4	38.9±24.9	31.3
B6	9.00±3.16	8	12.7±9.4	10.6
CD1	4.60±4.45	4	30.9±43.2	26.2

Aca23-WT and CD1 animals exhibited the greatest amount of axon loss (median 31% and 26%, respectively). B6 nerves had a median of 11% loss, while Aca23 mutant animals showed no significant axon loss (median=3% greater than control axon count; Table 6). The axon loss was significantly affected by mouse strain (Kruskal-Wallis, p=0.05). Aca23-WT axon loss was significantly greater than that in Aca23 (p=0.02). B6 had less axon loss than CD1 mice, but the difference was not significant with the sample size of this study (p=1.00). In previous published data, using larger sample sizes, B6 mice were significantly less susceptible to axon loss than CD1 mice [35].

## Discussion

Our findings represent the most detailed study to date of the structure of the sclera in experimental glaucoma in mice, demonstrating features that are presumed to be related to the susceptibility to glaucoma damage. The data suggest that the dynamic response of the sclera to chronic elevated IOP is as important as the baseline state of the tissue. This can be illustrated by comparing how susceptibility relates to baseline axial length and to change in axial length after glaucoma exposure. One simple hypothesis is that larger eyes would suffer greater scleral strain and more RGC damage at the same IOP as smaller eyes, all other things being equal. Indeed, myopia [39] is a well-established risk factor for open-angle glaucoma in humans and myopic eyes often have longer axial length and decreased stiffness [40-42]. Yet, the present data and our prior publications [5,35] showed that baseline axial length in the mouse is not a dominant glaucoma risk factor, since two mouse strains that are quite resistant to damage, B6 and Aca23, are at opposite ends of the axial length spectrum—B6 being quite small and Aca23 much longer and wider. Furthermore, the CD1 strain is highly susceptible to glaucoma injury and is intermediate in baseline length between B6 and Aca23.

The change in axial length with chronic IOP elevation seems more linked to degree of damage. Axial elongation also occurs with experimental glaucoma in rat, monkey [43], and infant human [44,45] eyes with chronic IOP elevation. The Aca23 mutant mice are more resistant to damage than their WT counterparts and have a longer axial length at baseline, but undergo less elongation with high IOP exposure. Similarly, the susceptible CD1 strain develops greater proportionate elongation than the more resistant B6 strain. Thus, the lack of change in eye length is more closely linked to lower glaucoma susceptibility than the baseline eye length.

The thickness of peripapillary sclera was different in fresh compared to fixed sclera, since aldehyde fixation and dehydration for embedding by organic solvents removes water and water-soluble, nonfibrillar components of the scleral extracellular matrix, including proteoglycans. Thus, the unfixed sclera was thicker than fixed sclera by 68% in control B6 and CD1 mice. Yet, in glaucoma eyes, the difference between the fresh and fixed sclera was lower than 10%. The lack of the normal difference between fresh and fixed thickness indicates that the glaucoma process may have reduced the nonfibrillar scleral components, namely water and proteoglycans. In fact, the fixed peripapillary scleral thickness in glaucomatous mouse eyes increased by 24% overall, indicating an expansion of the remaining fibrillar and cellular scleral components, which, while significant, was much smaller than the loss of water-soluble components. Thus, the difference between fresh and fixed scleral tissue responses suggests that the dynamic response of the sclera after glaucoma includes important shifts in composition, with soluble components decreasing and fibrillar components increasing in relative proportion. We are now conducting detailed proteomic analyses to confirm this hypothesis.

The microanatomy of the scleral lamellae indicates further dynamic changes that underlie glaucoma injury and differences among mouse strains that are associated with differential susceptibility. Both small-angle light scattering in tissue sections [19] and wide angle X-ray scattering in full-thickness [20] sclera show a circumferential pattern of fibrils in the peripapillary sclera and more random orientation in the mid-sclera. Our SHG observations of the peripapillary sclera showed that mouse eyes with substantial RGC axon loss undergo widening of the scleral canal, as seen in glaucomatous human eyes studied by scanning electron microscopy [46]. At a more detailed level, the measured number of lamellae increased by 18% in glaucomatous mice. Hypothetically, this could be due to a disruption in normal lamellae, leading to more of them being measured, or it could represent a rearrangement of lamellar orientation. We favor the latter explanation. After glaucoma induction, Aca23 mutants had higher numbers of cross-section (anteroposterior) lamellae and fewer longitudinal (circumferential) lamellae than their Aca23-WT littermates. SHG images show scleral lamellae interweaving in depth, so the shift toward cross-section lamellae may be a protective response to reorient lamellar orientation in the direction of greater stress, since we find that globe length increases more than globe width in experimental mouse glaucoma. The response of a woven structure of lamellae could produce scleral elongation by having the up and down direction of passage over and under adjacent lamellae pulled straighter (into antero-posterior alignment). In experimental myopia, the pattern of lamellar interweaving similarly reorients along the axis of elongation [42].

There was an increased thickness of each of the three categories (orientations) of collagen lamellae, but no significant change in density of fibrils. This suggests that the state of the sclera after glaucoma is more robust rather than weakened. Indeed, in B6, CD1, and Aca23 mice with chronic glaucoma [5], there was an increased stiffness with mechanical testing. It is not yet definitively established whether the greater stiffness of glaucomatous human eyes is present at baseline, whether it develops as a response to the disease, or both. The animal models indicate that chronic IOP elevation is associated with greater stiffness.

The diameter distribution of individual collagen fibrils was significantly different between glaucoma and normal mice, with significantly more small fibrils in the glaucoma eyes and fewer larger fibrils. This could occur by one of several mechanisms. First, atomic force microscopy shows that individual collagen fibrils subjected to stress may elongate by a tertiary structure change or as collagen molecules assembled within a fibril separate more than normal (enlarging the D-period) [47,48]. Second, there could be sliding of collagen molecules against each other within a fibril, potentially mediated by proteoglycan interactions [49]. Third, smaller fibrils could result from degradation or enzymatic alteration. Fourth, there may be new synthesis of fibrils smaller than the existing ones. The Aca23 mutant mice, that are most resistant to damage, had the largest increase in small collagen fibrils among strains studied. This suggests that it is neuroprotective to have more remodeling, or more elongation of collagen, or more new synthesis.

The number and volume of lamellae made up of scleral cells increased over all strains in peripapillary glaucoma sclera. Glaucoma Aca23 mutants had higher numbers of cellular lamellae than their Aca23-WT littermates at baseline. Furthermore, the expansion within each cellular layer with glaucoma was composed of increased intracellular volume by TEM. There are dramatic phenotypic alterations in scleral fibroblasts with mouse glaucoma that we detail in a companion report (personal communication, Ericka Oglesby). In addition, experimental glaucoma is associated with an upregulation of intracellular proteins involved in actin-, myosin-, and integrin-linked signaling. The specific transition from fibroblast to myofibroblast phenotype has been observed in experimental myopia [50], and is supported by our evidence in terms of experimental glaucoma. Scleral fibroblasts are likely to be contributory to responses to mechanical stress.

This work has several limitations. The mouse glaucoma model produces damage in weeks, compared to the human disease, which has a longer time frame. While general scleral structure is similar in mouse and man, the overall size of the human eye is 10 times larger. The present work was carried out using mostly younger mice, while human glaucoma is predominately a disease of older persons. We have found that, among mouse strains, older age can be either beneficial or detrimental to glaucoma susceptibility [35].

In summary, there is a decrease in the soluble compartment of the sclera and a vigorous response in its fibrillar component, including increased thickness and number of fibrillar lamellae. A shift to more lamellae oriented anteroposteriorly in posterior sclera indicates short-term plasticity in the interwoven framework of collagen groupings in response to elevated IOP. Both of these changes are consistent with the increased stiffness we have measured in globe inflation studies of glaucoma eyes. Localized retraction of the peripapillary scleral canal was detected with chronic IOP elevation. Expansion of the cellular scleral component with glaucoma points to the potential importance of the role of fibroblasts and their transition to myofibroblasts. Mice that are more resistant to glaucoma damage exhibit greater numbers of cellular lamellae at baseline, and with chronic IOP elevation, they have less globe elongation and a shift toward smaller collagen fibril diameter. The scleral response to glaucoma may be more important than its baseline state. Detailed study of this response is likely to provide clues to differential sensitivity to RGC loss in human eyes.

## Appendix 1.

Sclera Thickness, Number of Lamellae and Percent Increase after Glaucoma in different strains of mice (CD1, B6, Aca23-WT, and Aca23), at three different locations (peripapillary, nasal and temporal). To access the data, click or select the words “Appendix 1.” n=4–5 eyes in each category (fixed tissue); mean + standard deviation. *p<0.05, *t* test, †p<0.01, *t* test

## Appendix 2.

Orientation/ cellular component percent represented To access the data, click or select the words “Appendix 2.” Stacked columns represent the percent each orientation/ cellular component makes up for a given mouse strain and treatment group, in the peripapillary area. Fibrils in cross section are represented in red, those in longitudinal orientation are represented in green, fibrils in an oblique angle are represented in purple and cellular components are in blue

## References

[1] Anderson DR, Hendrickson A (1974). Effect of intraocular pressure on rapid axoplasmic transport in monkey optic nerve.. Invest Ophthalmol.

[2] Quigley HA, Addicks EM, Green WR, Maumenee AE (1981). Optic nerve damage in human glaucoma,II. The site of injury and susceptibility to damage.. Arch Ophthalmol.

[3] Foster CS, Sainz de la Maza M. The Sclera, Chapter 1: Structural Considerations of the Sclera. Springer Science Business Media, LLC; 2012; 1–30.

[4] Rada JAS, Shelton S, Norton TT (2006). The sclera and myopia.. Exp Eye Res.

[5] Nguyen C (2013). *, Cone FE***,** Nguyen TD, Coudrillier B, Pease ME, Steinhart MR, Oglesby EN, Quigley HA. Studies of Scleral Biomechanical Behavior Related to Susceptibility for Retinal Ganglion Cell Loss in Experimental Mouse Glaucoma.. Invest Ophthalmol Vis Sci.

[6] Downs JC, Blidner RA, Bellezza AJ, Thompson HW, Hart RT, Burgoyne CF (2002). Peripapillary scleral thickness in perfusion-fixed normal monkey eyes.. Invest Ophthalmol Vis Sci.

[7] Olsen TW, Aaberg SY, Geroski DH, Edelhauser HF (1998). Human sclera: thickness and surface area.. Am J Ophthalmol.

[8] Norman RE, Flanagan JG, Rausch SM, Sigal IA, Tertinegg I, Eilaghi A, Portnoy S, Sled JG, Ethier CR (2010). Dimensions of the human sclera: thickness measurement and regional changes with axial length.. Exp Eye Res.

[9] Quigley HA, Dorman-Pease ME, Brown AE (1991). Quantitative study of collagen and elastin of the optic nerve head and sclera in human and experimental monkey glaucoma.. Curr Eye Res.

[10] Hogan MJ, Alvarado JA, Weddell JE. Histology of the Human Eye.Philadelphia: W. B. Saunders Co. 1971, pp. 193–200.

[11] Anderson DR (1969). Ultrastructure of human and monkey lamina cribrosa and optic nerve head.. Arch Ophthalmol.

[12] Clark SJ, Keenan TDL, Fielder HL, Collinson LJ, Holley RJ, Merry CLR, van Kuppevelt TH, Day AJ, Bishop PN (2011). Mapping the differential distribution of glycosaminoglycans in the adult human retina, choroid, and sclera.. Invest Ophthalmol Vis Sci.

[13] Rada JA, Achen VR, Perry CA, Fox PW (1997). Proteoglycans in the human sclera—evidence for the presence of aggrecan.. Invest Ophthalmol Vis Sci.

[14] Winkler M, Chai D, Kriling S, Nien CJ, Brown DJ, Jester B, Juhasz T, Jester JV (2011). Nonlinear optical macroscopic assessment of 3-D corneal collagen organization and axial biomechanics.. Invest Ophthalmol Vis Sci.

[15] Thale A, Tillmann B, Rochels R (1996). Scanning electron-microscopic studies of the collagen architecture of the human sclera–normal and pathological findings.. Ophthalmologica.

[16] Meek KM, Fullwood NJ (2001). Corneal and scleral collagens—a microscopist’s perspective.. Micron.

[17] Hernandez MR, Luo XX, Igoe F, Neufeld AH (1987). Extracellular matrix of the human lamina cribrosa.. Am J Ophthalmol.

[18] Quigley HA, Brown A, Dorman-Pease ME (1991). Alterations in elastin of the optic nerve head in human and experimental glaucoma.. Br J Ophthalmol.

[19] Yan D, McPheeters S, Johnson G, Utzinger U, Vande Geest JP (2011). Microstructural differences in the human posterior sclera as a function of age and race.. Invest Ophthalmol Vis Sci.

[20] Pijanka JK, Coudrillier B, Ziegler K, Sorensen T, Meek KM, Nguyen TD, Quigley HA, Boote C (2012). Quantitative mapping of collagen fiber orientation in non-glaucoma and glaucoma posterior human sclerae.. Invest Ophthalmol Vis Sci.

[21] Gelman S, Cone FE, Pease ME, Nguyen TD, Myers K, Quigley HA (2010). The presence and distribution of elastin in the posterior and retrobulbar regions of the mouse eye.. Exp Eye Res.

[22] Girard MJA, Dahlmann-Noor A, Rayapureddi S, Bechara JA, Bertin BME, Jones H, Albon J, Khaw PT, Ethier CR (2011). Quantitative mapping of scleral fiber orientation in normal rat eyes.. Invest Ophthalmol Vis Sci.

[23] Girard MJA, Suh J-KF, Bottlang M, Burgoyne CF, Downs JC (2009). Scleral biomechanics in the aging monkey eye.. Invest Ophthalmol Vis Sci.

[24] McBrien NA, Cornell LM, Gentle A (2001). Structural and ultrastructural changes to the sclera in a mammalian model of high myopia.. Invest Ophthalmol Vis Sci.

[25] Wiesel TN, Raviola E (1977). Myopia and eye enlargement after neonatal lid fusion in monkeys.. Nature.

[26] Coudrillier B, Tian J, Alexander S, Myers KM, Quigley HA, Nguyen TD (2012). Biomechanics of the human posterior sclera: age- and glaucoma-related changes measured using inflation testing.. Invest Ophthalmol Vis Sci.

[27] Backhouse S, Phillips JR (2010). Effect of induced myopia on scleral myofibroblasts and in vivo ocular biomechanical compliance in the guinea pig.. Invest Ophthalmol Vis Sci.

[28] McBrien NA, Lawlor P, Gentle A (2000). Scleral remodeling during the development of and recovery from axial myopia in the tree shrew.. Invest Ophthalmol Vis Sci.

[29] Tejedor J, de la Villa P (2003). Refractive changes induced by form deprivation in the mouse eye.. Invest Ophthalmol Vis Sci.

[30] Burgoyne CF, Downs JC, Bellezza AJ, Suh J-KF, Hart RT (2005). The optic nerve head as a biomechanical structure: a new paradigm for understanding the role of IOP-related stress and strain in the pathophysiology of glaucomatous optic nerve head damage.. Prog Retin Eye Res.

[31] Cone FE, Gelman SE, Son JL, Pease ME, Quigley HA (2010). Differential susceptibility to experimental glaucoma among 3 mouse strains using bead and viscoelastic injection.. Exp Eye Res.

[32] Tamura Y, Konomi H, Sawada H, Takashima S, Nakajima A (1991). Tissue distribution of type VIII collagen in human adult and fetal eyes.. Invest Ophthalmol Vis Sci.

[33] Steinhart MR (2012). *, Cone FE*, Nguyen C, Nguyen TD, Pease ME, Puk O, Graw J, Oglesby E, Quigley HA. Mice with an induced mutation in Collagen 8A2 develop larger eyes and are resistant to retinal ganglion cell damage in an experimental glaucoma model.. Mol Vis.

[34] Puk O, Dalke C, Calzada-Wack J, Ahmad N, Klaften M, Wagner S, de Angelis MH, Graw J (2009). Reduced corneal thickness and enlarged anterior chamber in a novel ColVIIIa2G257D mutant mouse.. Invest Ophthalmol Vis Sci.

[35] Cone FE, Steinhart MR, Oglesby EN, Kalesnykas G, Pease ME, Quigley HA (2012). The effects of anesthesia, mouse strain and age on intraocular pressure and an improved murine model of experimental glaucoma.. Exp Eye Res.

[36] Levkovitch-Verbin H, Quigley HA, Martin KR, Zack DJ, Pease ME, Valenta DF (2003). A Model to Study Differences between Primary and Secondary Degeneration of Retinal Ganglion Cells in Rats by Partial Optic Nerve Transection.. Invest Ophthalmol Vis Sci.

[37] Freund I, Deutsch M (1986). Sprecher. Connective tissue polarity; Optical second- harmonic microscopy, crossed- beam summation and small- angle scattering in rat-tail tendon.. Biophys J.

[38] Zipfel WR, Williams RM, Christie R, Nikitin AY, Hyman BT, Webb WW (2003). Live tissue instrinsic emission microscopy using multiphoton-excited native fluorescence and second harmonic generation.. Proc Natl Acad Sci USA.

[39] Boland MV, Quigley HA (2007). Risk factors and open-angle glaucoma: concepts and applications.. J Glaucoma.

[40] Curtin BJ (1969). Physiopathologic aspects of scleral stress-strain.. Trans Am Ophthalmol Soc.

[41] Curtin BJ, Teng CC (1958). Scleral changes in pathological myopia.. Trans Am Acad Ophthalmol Otolaryngol.

[42] McBrien NA, Jobling AI, Gentle A (2009). Biomechanics of the sclera in myopia: Extracellular and cellular factors.. Optom Vis Sci.

[43] Yang H, Thompson H, Roberts MD, Sigal IA, Downs JC, Burgoyne CF (2011). Deformation of the early glaucomatous monkey optic nerve head connective tissue after acute IOP elevation in 3-D histomorphometric reconstructions.. Invest Ophthalmol Vis Sci.

[44] Quigley HA (1977). The pathogenesis of reversible cupping in congenital glaucoma.. Am J Ophthalmol.

[45] Maumenee AE (1962). Further observations on the pathogenesis of congenital glaucoma.. Trans Am Ophthalmol Soc.

[46] Quigley HA, Hohman RM, Addicks EM, Massof RS, Green WR (1983). Morphologic changes in the lamina cribrosa correlated with neural loss in open-angle glaucoma.. Am J Ophthalmol.

[47] Rigozzi S, Stemmer A, Müller R, Snedeker JG (2011). Mechanical response of individual collagen fibrils in loaded tendon as measured by atomic force microscopy.. J Struct Biol.

[48] Gautieri A, Vesentini S, Redaelli A, Buehler MJ (2011). Hierarchical structure and nanomechanics of collagen microfibrils from the atomistic scale up.. Nano Lett.

[49] Rigozzi S, Muller R, Stemmer A, Snedeker JG (2013). Tendon glycosaminoglycan proteoglycan side chains promote collagen fibril sliding—AFM observations at the nanoscale.. J Biomech.

[50] Phillips JR, McBrien NA (2004). Pressure-induced changes in axial eye length of chick and tree shrew: Significance of myofibroblasts in the sclera.. Invest Ophthalmol Vis Sci.

